# A comparison of methods to calculate a total merit index using stochastic simulation

**DOI:** 10.1186/s12711-015-0118-4

**Published:** 2015-05-02

**Authors:** Christina Pfeiffer, Birgit Fuerst-Waltl, Hermann Schwarzenbacher, Franz Steininger, Christian Fuerst

**Affiliations:** Department of Sustainable Agricultural Systems, Division of Livestock Sciences, University of Natural Resources and Life Sciences Vienna, Gregor-Mendel-Straße 33, 1180 Vienna, Austria; ZuchtData EDV-Dienstleistungen GmbH, Dresdner Straße 89/19, 1200 Vienna, Austria

## Abstract

**Background:**

Modern dairy cattle breeding goals include several production and more and more functional traits. Estimated breeding values (EBV) that are combined in the total merit index usually come from single-trait models or from multivariate models for groups of traits. In most cases, a multivariate animal model based on phenotypic data for all traits is not feasible and approximate methods based on selection index theory are applied to derive the total merit index. Therefore, the objective of this study was to compare a full multitrait animal model with two approximate multitrait models and a selection index approach based on simulated data.

**Methods:**

Three production and two functional traits were simulated to mimic the national Austrian Brown Swiss population. The reference method for derivation of the total merit index was a multitrait evaluation based on all phenotypic data. Two of the approximate methods were variations of an approximate multitrait model that used either yield deviations or de-regressed breeding values. The final method was an adaptation of the selection index method that is used in routine evaluations in Austria and Germany. Three scenarios with respect to residual covariances were set up: residual covariances were equal to zero, or half of or equal to the genetic covariances.

**Results:**

Results of both approximate multitrait models were very close to those of the reference method, with rank correlations of 1. Both methods were nearly unbiased. Rank correlations for the selection index method showed good results when residual covariances were zero but correlations with the reference method decreased when residual covariances were large. Furthermore, EBV were biased when residual covariances were high.

**Conclusions:**

We applied an approximate multitrait two-step procedure to yield deviations and de-regressed breeding values, which led to nearly unbiased results. De-regressed breeding values gave even slightly better results. Our results confirmed that ignoring residual covariances when a selection index approach is applied leads to remarkable bias. This could be relevant in terms of selection accuracy. Our findings suggest that the approximate multitrait approach applied to de-regressed breeding values can be used in routine genetic evaluation.

## Background

In dairy cattle breeding programs, selection is focused on different production traits and more and more on functional traits. Usually, estimated breeding values (EBV) or phenotypes [1] are combined into a total merit index (TMI) [2]. Traits or groups of traits are usually evaluated separately based on different statistical models [3]. This is also the case in the joint Austrian-German genetic evaluation of one dairy and several dual-purpose cattle breeds. The TMI and several sub-indices are based on a selection index method [1] for all cattle breeds, which was proposed by [4]. Currently, the TMI of Fleckvieh (dual-purpose Simmental) and Brown Swiss populations consists of up to 30 production and functional traits. EBV for the TMI as well as for several sub-indices are estimated either using univariate (e.g. protein yield) or multivariate (e.g. calving ease and stillbirth) methods by applying animal or sire-maternal-grandsire models (the latter for functional longevity only). Some of these models include repeated measures, such as somatic cell count [2]. Subsequently, EBV for individual traits are combined to form TMI or other sub-indices by assuming that residual covariances between traits or groups of traits are equal to zero. Due to the large number of traits involved and to methodological constraints, the additive genetic (co)variance matrix to combine EBV into a TMI cannot be estimated multivariately for all traits. Additive genetic correlations between the traits in the TMI are mainly obtained from the literature [4]. Furthermore, we have observed that the TMI shows an upward trend in bias for animals with low reliabilities (r^2^) (unpublished results). We hypothesize that this is due to ignoring residual covariances when combining traits or trait groups into the TMI. Full multivariate estimation of all traits based on phenotypic data could represent the optimum methodology [5-7], but in routine genetic evaluations substantial restrictions (e.g. computer power and computational considerations) make this approach infeasible. Thus, an approximate multivariate model using a two-step procedure was proposed and validated using simulated data [3,8,9]. In the first step, genetic (co)variances were estimated based on yield deviations (YD). The associated weights for YD were calculated from univariate analyses and YD were adjusted for all environmental effects. In the second step, a multivariate animal model that included a random genetic effect for the animal and a fixed year effect was applied [3,9]. Due to improved connectedness of the data and simultaneous estimation of genetically correlated traits, accuracies of EBV can be increased compared to those from a univariate analysis. In particular, functional traits, which are characterized by low heritabilities (h^2^), benefit more from a multivariate animal model than moderately to highly heritable traits [6,10]. A set of characteristics using an approximate multivariate model based on YD exists and is well described [3,7,11,12]. However, YD cannot always be obtained in routine evaluation settings, e.g. for EBV from Interbull. Therefore, the objective of this study was to compare four methods to calculate TMI using: a full multitrait model; approximate multitrait models applied to YD and de-regressed estimated breeding values (drEBV); and the current selection index method which is used in the joint Austrian-German genetic evaluations. The consequences of ignoring residual covariances when a TMI is computed were investigated. Hence, three scenarios with respect to residual covariances were set up using data that were generated by stochastic simulation of data that represent a simplified breeding scheme of the Austrian Brown Swiss cattle population.

## Methods

### Data simulation

A population structure that roughly reflected the Austrian breeding program of Brown Swiss cattle was simulated with the stochastic simulation program ADAM [13]. The simulated population comprised approximately 51 300 cows in 1710 herds. Five normally distributed traits were chosen to represent dairy, beef and fitness traits. Additional requirements were a wide range of heritabilities and genetic correlations between traits and economic importance. Fat yield (FY), protein yield (PY), somatic cell count (SCC) and non-return rate of cows (NRR) were measured on female animals. Net daily gain (NDG) was observed on approximately 60% of all male animals. No repeated records were assumed. The assumed heritabilities and genetic correlations for the five traits are in Table 1, which were obtained from estimates for the national Brown Swiss population [14]. Around 25% young bulls and 75% proven bulls were used for matings in the selection scheme. Breeding values and phenotypes for the five traits were simulated for animals from a base population. Animals were selected on a TMI based on multivariately EBV over 30 years. Based on values used for routine genetic evaluation, relative economic weights per additive genetic standard deviation were 5.4, 53.6, 4.3, 19.7 and 17% for FY, PY, NDG, SCC and NRR, respectively [14]. Three scenarios with respect to residual covariances between traits were simulated. In scenario 0, residual correlations between traits were assumed to be zero. For scenarios 1 and 2, residual correlations corresponded to 50% or 100% of the genetic correlations, respectively. These scenarios were chosen to specifically test the currently used selection index method for possible bias due to ignoring correlations between residual effects, as proposed by [4]. For each scenario, ten replicates were simulated. On average, breeding values and reliabilities of EBV were estimated for 755 567 animals for each scenario.

**Table 1:** **Genetic parameters used for simulation (heritabilities on the diagonal, genetic correlations above the diagonal)**

**Trait**	**FY**	**PY**	**NDG**	**SCC**	**NRR**
FY	0.40	0.85	0.10	0.25	−0.20
PY		0.39	0.10	0.25	−0.20
NDG			0.27	0.00	0.00
SCC				0.12	−0.10
NRR					0.02

Fat yield (FY), protein yield (PY), net daily gain (NDG), somatic cell count (SCC) and non return rate cow (NRR).

### Methods to calculate the TMI

The first method (MULTI) was a full multivariate animal model based on phenotypic data using the true genetic and phenotypic parameters, assuming the following linear model:

1 $$ \mathbf{y} = \mathbf{X}\mathbf{b} + \mathbf{Z}\mathbf{a} + \mathbf{e}, $$

where **y** is the vector representing simulated phenotypic observations of FY, PY, NDG, SCC and NRR, respectively; **X** and **Z** represent the incidence matrices for fixed and random effects, respectively, **b** is the vector of the fixed herd-year effects, **a** is the vector of the random animal additive genetic effects and **e** is the vector of the random residual effects. Vector **a** was assumed to have a multivariate normal distribution, with MVN(0, **G** = **G**_**0**_ ⊗ **A)**, where **G**_**0**_ is a 5 × 5 additive genetic variance-covariance matrix,⊗ is the Kronecker product of matrices, and **A** represents the numerator relationship matrix. Residuals **e** were assumed to be MVN(0, **R = R**_**0**_ ⊗ **I**), where **R**_**0**_ is the 5 × 5 residual variance-covariance matrix and **I** represents the identity matrix. Subsequently, the TMI was calculated as:

2 $$ {\mathrm{TMI}}_{\mathrm{MULTI}}={\widehat{\mathrm{a}}}_{\mathrm{FY}}{\upomega}_{\mathrm{FY}}+{\widehat{\mathrm{a}}}_{\mathrm{PY}}{\upomega}_{\mathrm{PY}}+{\widehat{\mathrm{a}}}_{\mathrm{NDG}}{\upomega}_{\mathrm{NDG}}+{\widehat{\mathrm{a}}}_{\mathrm{SCC}}{\upomega}_{\mathrm{SCC}}+{\widehat{\mathrm{a}}}_{\mathrm{NRR}}{\upomega}_{\mathrm{NRR}}, $$

where â represents the EBV for the traits and ω denotes the relative economic values. As the full multitrait estimation, MULTI, represents the optimum method, this was considered to be the reference method.

The second (YD) and third (DRP) methods were based on the approximate multitrait two-step procedure proposed by [3]. These methods use either YD or drEBV. For both methods, univariate genetic evaluations were used to calculate YD and drEBV for each trait. For the YD method, phenotypic observations were corrected for the fixed herd-year effect using the following model:

3 $$ \mathbf{y}* = \mathbf{y}\ \hbox{--}\ \mathbf{X}\mathbf{b}, $$

where y* is the vector of yield deviations, **y** is the vector of phenotypic observations of the traits FY, PY, NDG, SCC and NRR, and **b** is the vector of herd-year effects. **X** is the incidence matrix of proper order. After correcting for fixed effects, all five traits were analysed together in the following multivariate animal model:

4 $$ \mathbf{y}* = \mathbf{X}\mathbf{b} + \mathbf{Z}\mathbf{a} + \mathbf{e}, $$

where y* indicates yield deviations of animals of a trait; **X** and **Z** represent the incidence matrices for fixed and random effects, respectively, **b** is the vector of year of birth fixed effects, **a** is the vector of random animal additive genetic effects and **e** denotes the vector of random residual effects. The year of birth effect was included in order to account for over- or underestimation of the genetic trend [9]. The use of YD requires weights to consider the different amount of information for each animal. Reliabilities were calculated with the program package ApaX [15], using the approximate Interbull method of [15]. Based on these reliabilities, effective own performances (EOP) were calculated and used as weighting factors for yield deviations in the multivariate estimation of breeding values for method YD. The following formula was applied to calculate EOP for trait i:

5 $$ {\mathrm{EOP}}_{\mathrm{i},\mathrm{j}}=\frac{\upalpha_{\mathrm{i}}}{1\hbox{-} {\mathrm{r}}_{\mathrm{i},\mathrm{j}}^2}-{\upalpha}_{\mathrm{i},} $$

where α_i_ is the ratio of residual and additive genetic variances of trait i; and r^2^ represents the reliability of the own performance of animal j for trait i [16]. The assumption and size of the (co)variance matrices **G**_**0**_ and **R**_**0**_ for estimating breeding values and the equation for the TMI were based on the same formulas as in method MULTI. For method DRP, EBV were de-regressed using a univariate de-regression based on the approach of [17] and [18], which is implemented in the program package MiX99 [19]. The de-regression procedure uses the EBV and their respective effective daughter contributions as weights, with the general mean as the only fixed effect. Based on model Equation (4), EBV were estimated in a 5-trait animal model, with drEBV as the response variables and an overall mean, a year effect, additive genetic effects of animals for each trait and residual effects of drEBV as explanatory variables. As described for method YD, EOP were used as weighting factors to estimate breeding values.

The fourth method SI is the approach that is currently used in routine genetic evaluations in Austria and Germany to calculate the total merit index based on selection index theory. The EBV of the five traits were estimated univariately using the same model Equation (1) but assuming that **G = A**${\upsigma}_{\mathrm{a}}^2$ and **R = I**${\upsigma}_{\mathrm{e}}^2$, where ${\upsigma}_{\mathrm{a}}^2$ is the additive genetic variance and ${\upsigma}_{\mathrm{e}}^2$ is the residual variance. In order to obtain the TMI with method SI, covariances between EBV (σ_ij_) were calculated as:

6 $$ {\upsigma}_{\mathrm{i}\mathrm{j}} = {\mathrm{r}}_{\mathrm{gij}}{{\mathrm{r}}^2}_{\mathrm{i}}{{\mathrm{r}}^2}_{\mathrm{j}}{\upsigma}_{\mathrm{ai}}{\upsigma}_{\mathrm{aj}}, $$

where r_gij_ is the genetic correlation between traits i and j; r^2^_i,j_ are the reliabilities of EBV of traits i and j, and σ_ai,aj_ are the additive genetic standard deviations of traits i and j. In this equation, residual correlations are neglected, i.e. assumed to be zero.

YD, drEBV and EBV were computed using the program package MiX99 [19]. Reliabilities of EBV were calculated with the program package ApaX [15]. Reliabilities of the TMI of methods MULTI, YD and DRP were calculated using the approach of [20]. Reliabilities of the TMI of method SI were calculated using the formula described by [4]. For all methods, genetic parameters were not re-estimated, but the true (simulated) parameters were used. All EBV were standardized to an additive genetic standard deviation of 12 and 100 for the mean of cohorts from birth year 18 to 22. Spearman rank correlations between TMI of different methods were calculated using SAS 9.2. [21]. To estimate breeding values and reliabilities by calculating genetic trends and for all further analyses, the last 20 years of the simulated period were used.

## Results

### Correlations

Table 2 shows the Spearman rank correlations between TMI obtained using the reference method MULTI and from methods YD, DRP and SI categorized by TMI reliability for all animals for scenarios 0 and 2. Correlations for YD and especially DRP were almost 1. Correlations between the reference method and method SI were slightly lower, particularly for scenario 2, in which the residual (and thus the phenotypic) correlation was equal to the genetic correlation. Results for scenario 1 are not shown since values were always between those of scenarios 0 and 2. The simulated selection program resulted in a strong genetic trend, therefore results were also analyzed within year groups. Across all animals and in the last 20 years of the simulation (Table 3), correlations between TMI from the different methods were high and ranged from 0.983 to 1 for all scenarios. As expected, correlations decreased slightly when split into year groups. Correlations for method SI were more strongly affected, especially when non-zero residual covariances were simulated. In addition, the population was subdivided into the following four groups: bulls with progeny information (BP), bulls without progeny information (BNP), females with progeny information (FP) and females without progeny information (FNP). Correlations between TMI from the reference method MULTI and the three other methods for BP, BNP, FP and FNP across all 20 years and scenarios ranged from 0.984 to 1 (results not shown). For bulls and dams with progeny, a tendency for higher correlations was observed. Method SI showed the lowest correlations and method DRP showed the highest correlations with method MULTI. With regard to scenarios, rank correlations were highest for scenario 0 followed by scenarios 1 and 2. When grouped by reliability, rank correlations of TMI from MULTI with those from YD and DRP were always close to 1. However, correlations between MULTI and SI grouped by reliability were lower, especially when non-zero residual correlations were simulated (scenarios 1 and 2, results not shown). For BNP bulls, correlations even declined to 0.907.

**Table 2:** **Rank correlations of TMI obtained using alternate methods with TMI obtained using the multivariate method within TMI reliability groups**

**Scenario**	**Reliability**	**YD**	**DRP**	**SI**
0	<39	1.000	1.000	0.993
	40-49	1.000	1.000	0.990
	50-59	1.000	1.000	0.997
	60-69	1.000	1.000	0.994
	70-79	1.000	1.000	0.991
	80-89	1.000	1.000	0.997
	>90	1.000	1.000	0.999
2	<39	1.000	1.000	0.988
	40-49	1.000	1.000	0.985
	50-59	1.000	1.000	0.988
	60-69	1.000	1.000	0.985
	70-79	1.000	1.000	0.978
	80-89	1.000	1.000	0.991
	>90	1.000	1.000	0.998

YD = approximate multitrait two-step procedure based on yield deviations; DRP = approximated multitrait two-step procedure based on de-regressed estimated breeding values; SI = selection index method.

**Table 3:** **Rank correlations with multivariate TMI (MULTI) within year groups for different TMI methods for scenarios 0 and 2**

**Scenario**	**Years**	**YD**	**DRP**	**SI**
0	All	1.000	1.000	0.989
	11-15	1.000	1.000	0.962
	16-20	1.000	1.000	0.963
	21-25	1.000	1.000	0.945
	26-30	1.000	1.000	0.950
2	All	1.000	1.000	0.983
	11-15	1.000	1.000	0.948
	16-20	1.000	1.000	0.943
	21-25	1.000	1.000	0.914
	26-30	1.000	1.000	0.932

YD = approximate multitrait two-step procedure based on yield deviations; DRP = approximated multitrait two-step procedure based on de-regressed estimated breeding values; SI = selection index method.

### Bias

**Figure 1:**
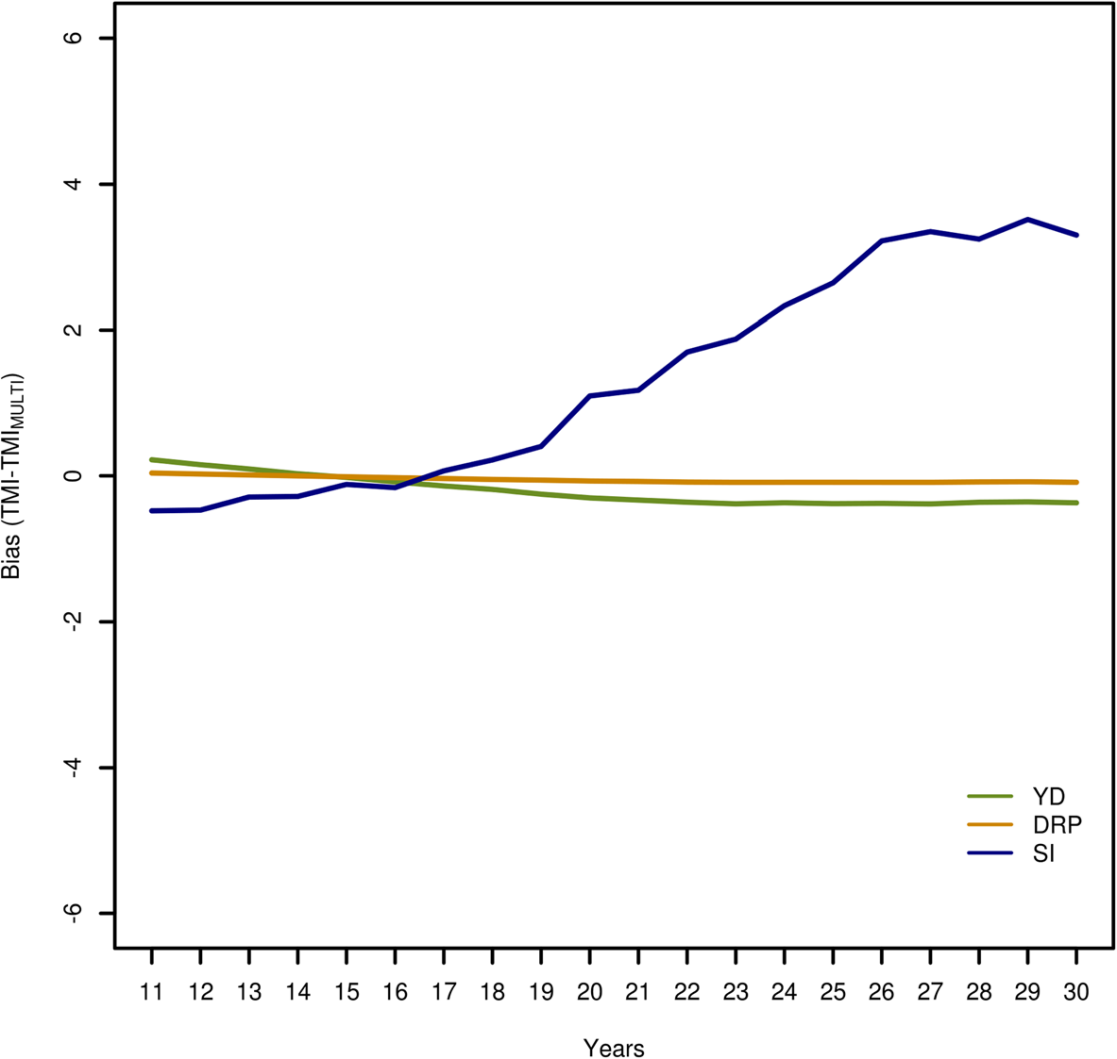
**Time trend of bias (TMI-TMI**_**MULTI**_ **) with different TMI methods for the top 10% animals per year in scenario 0.**

**Figure 2:**
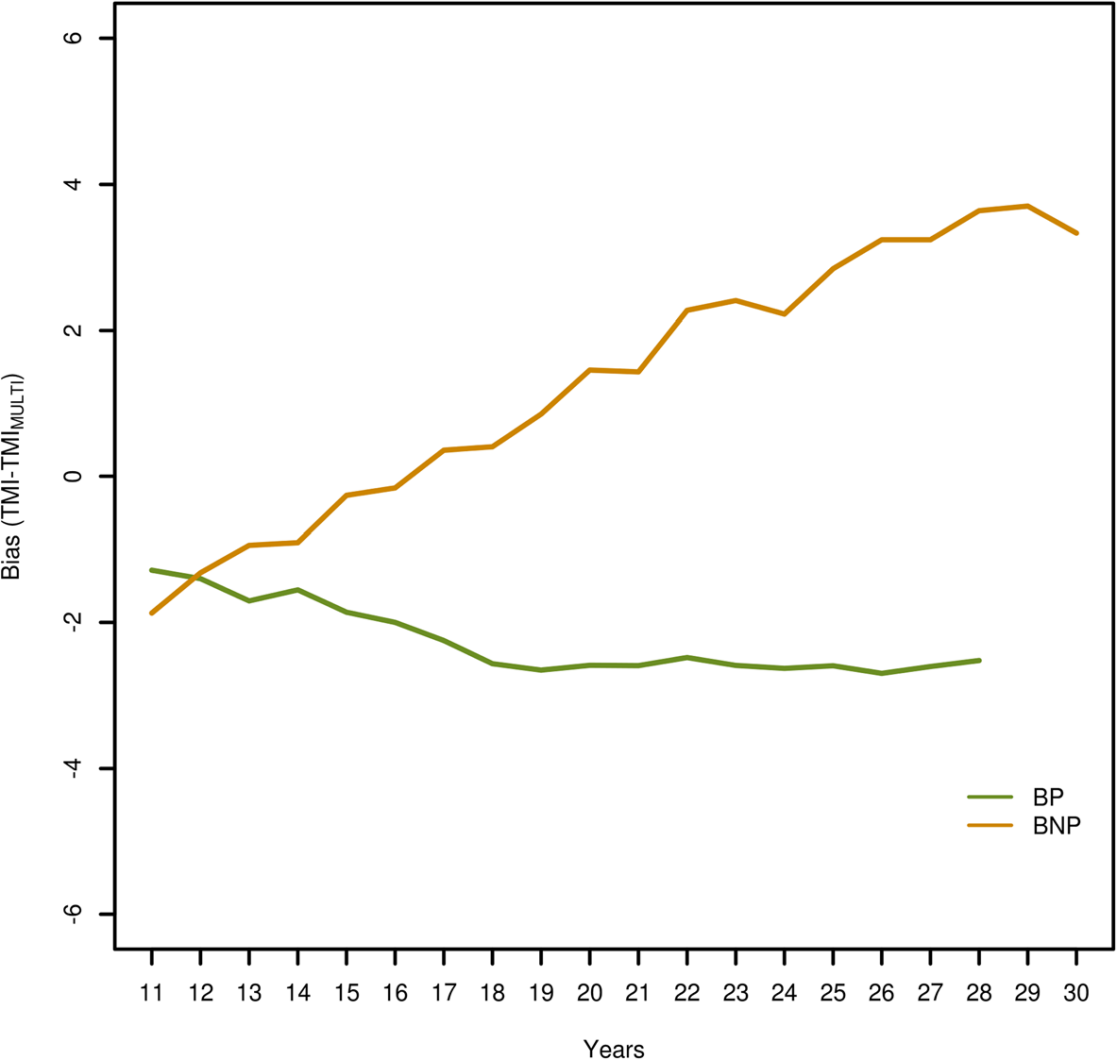
**Time trend of bias (TMI-TMI**_**MULTI**_ **) for bulls with progeny (BP) and bulls without progeny (BNP) (method SI, scenario 0).**

**Figure 3:**
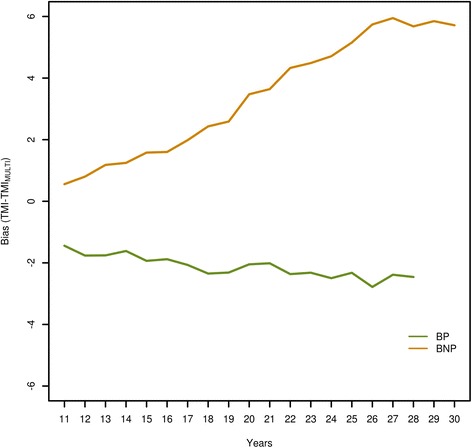
**Time trend of bias (TMI-TMI**_**MULTI**_ **) for the top 10% bulls with progeny (BP) and bulls without progeny (BNP) by year (method SI, scenario 0).**

Bias was defined as the difference between the estimated TMI of each method and the MULTI TMI (e.g. TMI_YD_-TMI_MULTI_). This was done for all animals and scenarios. Since the reference method MULTI essentially had no bias, the presented bias is equivalent to the bias from true TMI. Results for scenarios 0 and 2 for all animals grouped by reliability are in Table 4. For all scenarios, methods YD and DRP showed almost no bias. However, method SI showed a relevant bias in both scenarios. The TMI of animals with a reliability lower than 50% were overestimated, whereas that of animals with a reliability greater than 50% were underestimated. When residual correlations were non-zero (scenario 2), this bias was more pronounced. Method SI led to a markedly overestimated genetic trend, which was expressed as a downwards bias during the first years and an upwards bias during the last years (Table 5). This was especially evident for scenario 2 and even more for the top 10% animals based on TMI (Figure 1). When non-zero residual covariances were simulated (scenario 2), the bias was similar to that for scenario 0 but slightly more pronounced. Figure 2 shows the bias for all bulls with progeny performance (BP) and for bulls without progeny performance (BNP) for method SI for scenario 0 (currently used method). A difference of about half a genetic standard deviation was observed between the average TMI of BP and BNP at the end of the simulated period. Selecting the best 10% bulls each year from groups BP and BNP resulted in an even greater difference (Figure 3) for method SI. Differences between groups BP and BNP were even higher when the simulated residual covariances were greater (results are not shown). The difference between FP and FNP was close to 0 for method SI for all scenarios.

**Table 4:** **Bias of approximate TMI methods relative to TMI from multivariate analysis within TMI reliability groups for scenarios 0 and 2**

**Scenario**	**Reliability**	**YD**	**DRP**	**SI**
0	<39	0.1	0.0	1.0
	40-49	0.1	0.0	1.3
	50-59	0.1	0.0	−0.5
	60-69	0.1	0.0	−0.6
	70-79	−0.1	0.0	−1.6
	80-89	0.0	0.0	−2.3
	>90	0.0	0.0	−2.5
2	<39	0.1	0.0	0.2
	40-49	0.1	0.0	0.7
	50-59	0.1	0.0	−1.1
	60-69	0.1	0.0	−1.4
	70-79	−0.1	0.0	−1.6
	80-89	0.0	0.0	−2.6
	>90	0.0	0.0	−2.8

YD = approximate multitrait two-step procedure based on yield deviations; DRP = approximated multitrait two-step procedure based on de-regressed estimated breeding values; SI = selection index method.

**Table 5:** **Bias with different TMI methods from multivariate TMI within year groups for scenarios 0 and 2**

**Scenario**	**Years**	**YD**	**DRP**	**SI**
0	All	0.1	0.0	0.2
	11-15	0.4	0.1	−1.1
	16-20	0.1	0.0	−0.3
	21-25	−0.1	0.0	0.6
	26-30	−0.1	0.0	1.4
2	All	0.1	0.0	−0.5
	11-15	0.4	0.1	−2.7
	16-20	0.1	0.0	−0.7
	21-25	−0.1	0.0	0.7
	26-30	−0.1	0.0	0.8

YD = approximate multitrait two-step procedure based on yield deviations; DRP = approximated multitrait two-step procedure based on de-regressed estimated breeding values; SI = selection index method.

### Reliabilities and standard deviations

The mean reliabilities (r^2^) and standard deviations (SD) for the TMI of methods MULTI, YD and DRP were almost equal (numerically ±0.1 to ±0.3). Thus, only the results of the reference MULTI and SI methods are presented in Table 6. Estimated reliabilities were slightly higher for method SI than for the other methods for scenario 0, when grouped by years. For scenario 2, for which non-zero residual covariances were simulated, reliabilities for method SI were substantially higher than for MULTI for each year group. Since the estimated reliabilities depend on the method used, realised (true) reliabilities obtained from the squared correlation of estimated true breeding values for TMI are also presented. Realised reliabilities for SI were on average 4% lower than for MULTI.

**Table 6:** **Reliabilities and standard deviations (SD) of TMI from methods MULTI and SI for scenarios 0 and 2 grouped by year and loss in selection response (SR) with SI compared to MULTI**

**Sc.**	**Years**	**Reliabilities MULTI**		**Reliabilities SI**		**SD MULTI**		**SD SI**		**Loss in SR**
		**Realized**	**Estimated**	**Realized**	**Estimated**	Expected*	**Estimated**	Expected*	**Estimated**	**With SI** ^**#**^
**0**	11-15	31.3	46.1	27.9	48.8	5.7	5.7	5.4	6.0	−3.2
	16-20	32.1	45.9	28.8	48.6	5.7	5.9	5.4	6.1	−2.7
	21-25	35.4	46.0	31.2	48.8	6.1	6.2	5.7	6.7	−3.8
	26-30	42.0	45.7	38.0	48.3	6.9	7.2	6.6	7.9	−3.7
**2**	11-15	28.7	43.6	24.3	48.9	5.4	5.5	5.0	6.6	−4.6
	16-20	27.6	43.4	23.0	48.7	5.2	5.3	4.8	6.4	−4.8
	21-25	30.9	43.3	25.4	48.6	5.6	5.8	5.1	6.9	−5.2
	26-30	36.7	43.0	32.0	48.4	6.3	6.6	5.9	8.0	−3.9

Sc. = Scenario; *expected standard deviation of EBV, based on realised reliabilities, obtained from squared correlation of estimated with true breeding values, and the true genetic standard deviation within year; ^#^percent loss in SR of SI compared to MULTI, with 100% being a SR with an accuracy of 1.

To quantify the presumed inflation of TMI from method SI, the expected SD of TMI was calculated as, $\sqrt{{\mathrm{r}}^2\upsigma {\scriptscriptstyle \frac{2}{\mathrm{T}}}}$ using realised reliabilities and the true genetic standard deviation${\upsigma}_{\mathrm{T}}^2$within year (Table 6). The variance for TMI for method MULTI was very close to the expected values. For method SI, the observed SD of the TMI was notably higher than the expected SD for scenario 0 (from 0.4 to 1) and especially for scenario 2 (from 1.6 to 2.1). These findings were similar for all scenarios and groups (results not shown).

### Selection response

To demonstrate the practical implications of the lower realised reliabilities and the inflated SD of EBV with method SI, the expected loss in selection response using SI compared to MULTI was analysed. If we consider the breeder’s equation, we can assume that selection intensity, genetic standard deviation and generation interval do not depend on the TMI method. Thus, differences in selection response between the analysed methods depend only on the reliability of the TMI. This led to an expected loss in response of 3 to 5% with method SI compared to MULTI (Table 6). Since the realised reliabilities for methods YD and DRP were almost identical to those for MULTI, no loss in response is expected for these methods.

## Discussion

In this study, four methods were used to combine several traits into a total merit index and were compared with different assumptions regarding residual covariances. Both approximate multitrait two-step procedures that used either YD or drEBV led to results that were comparable to those of the full multivariate animal model (MULTI). Our findings agree with those of [8,9] who compared a full multitrait model, an approximate two-step procedure applied to YD and a combination of single trait models. The approximate two-step procedure was not as efficient as the full multitrait model but superior to the single-trait approach in terms of genetic response. Results of this study substantiate some drawbacks of the method that is currently used to calculate a TMI in routine genetic evaluations in Germany and Austria. Spearman rank correlations of TMI between the reference MULTI and the YD and DRP methods were close to 1 for all scenarios for any categories of reliability or year. Correlations for method SI were lower than for methods YD and DRP even when zero residual covariances were simulated. Rank correlations between MULTI and SI decreased with increasing residual covariances. Applying selection index theory (e.g. method SI) to calculate a TMI is valid when traits are not or only slightly correlated [3,12]. However, this is not the case for the routinely calculated TMI in Austria and Germany due to the wide range of dairy, beef and functional traits which are correlated to a certain degree (e.g. genetic correlations between fat kg and fertility, dressing percentage or milkability are -0.20, -0.15 and 0.25, respectively [14]). In particular, some of the lowly heritable functional traits are correlated with production or conformation traits to a considerable degree [12,22]. Furthermore, it is well known that ignoring residual covariances when animals are recorded for different traits in the same environment is not valid [3]. This was confirmed by our results with method SI. As residual covariances increased, correlations of TMI with the reference method decreased and deviated more from the full multivariate model. In addition, a downwards bias was observed for animals with high reliabilities, e.g. bulls with progeny, and an upwards bias for animals with low reliabilities, e.g. young animals without progeny. Hence, the bias for the top 10% animals can be relevant in terms of selection decisions across birth cohorts. The average difference between TMI from the reference MULTI and SI methods can be up to half a genetic SD, which leads to substantial re-ranking of bulls. This can cause selection bias, in particular when early selection decisions are made on young bulls. It should be noted that, in this simulation study, young bulls without progeny cannot be compared with young bulls with genomic EBV from routine evaluations. In the simulation, young bulls had own data only for NDG, while for all other traits a pedigree index was used. This means that the problem of bias may be less severe in routine evaluations.

In the method that is currently used to calculate a TMI, residual correlations are neglected for traits for which covariance is expected to occur, e.g. fat and protein yield, functional longevity and some fitness traits [22,23]. Results of this study based on simulated data imply that the variances of EBV with low reliability are in general inflated using method SI. This is confirmed by results obtained with real data on functional longevity and type traits in Austrian Fleckvieh cows [24]. Reliabilities and variances of the TMI were overestimated for functional longevity when combined with auxiliary type traits using method SI and ignoring residual covariances. Based on the results of this simulation, drEBV could be a good alternative to YD, since they can be easier to obtain in some cases and show equally good results. This could also help to include Interbull EBV in the national evaluation, since individual YD are not available at the international level. The current method for calculating TMI and other sub-indices could thus be replaced by an approximate two-step procedure using drEBV.

Two crucial points need to be clarified before the approximate multitrait approach can be implemented in routine genetic evaluations: (1) accurate genetic and residual (co)variance components must be estimated using a multivariate analysis of all traits in order to allow their inclusion in the two-step procedure; if this is not possible, it has been suggested by [8] to cluster traits that have genetic correlations above 0.10; and (2) furthermore, a genomic evaluation including international EBV (Interbull) must be implemented.

## Conclusions

An approximate multitrait two-step procedure to compute TMI applied to drEBV led to nearly unbiased results. Fortunately, the outcomes for the multitrait method based on drEBV are equally as good as those on YD, which will facilitate its implementation, especially for specific traits for which it is difficult to obtain accurate YD. The advantages of these methods are greatest when residual covariances differ from zero. Although there are several crucial prerequisites before implementing an approximate multitrait two-step procedure in routine genetic evaluations, our results open up perspectives for the replacement of the current selection index method by this procedure based on drEBV.
